# Oleandrin synergizes with cisplatin in human osteosarcoma cells by enhancing cell apoptosis through activation of the p38 MAPK signaling pathway

**DOI:** 10.1007/s00280-018-3692-7

**Published:** 2018-09-28

**Authors:** Lei Yong, Yunlong Ma, Bin Zhu, Xiao Liu, Peng Wang, Chen Liang, Guanping He, Zhigang Zhao, Zhongjun Liu, Xiaoguang Liu

**Affiliations:** 1 Department of Orthopedics, Peking University Third Hospital, No. 49, North Garden Street, Haidian District, Beijing, 100191 People’s Republic of China; 2 The Center for Pain Medicine, Peking University Third Hospital, No. 49, North Garden Street, Haidian District, Beijing, 100191 People’s Republic of China

**Keywords:** Osteosarcoma, Cisplatin, Oleandrin, Synergistic effect, P38 MAPK

## Abstract

**Purpose:**

Our previous studies have reported the antitumor effect of oleandrin on osteosarcoma; however, its chemosensitizing effect in osteosarcoma treatment is still unknown. Therefore, we explored the sensitizing effects of oleandrin to cisplatin in osteosarcoma and investigated the potential mechanisms.

**Methods:**

After exposure to oleandrin and/or cisplatin, CCK-8 and colony formation assays, DAPI staining and flow cytometry were performed to detect cell proliferation and apoptosis in 143B, U-2OS and MG-63 osteosarcoma cells. The median-effect analysis was applied to evaluate the combined effect. Western blot was used to determine the expression of related proteins. Osteosarcoma xenografts and histological observations were applied to confirm the combined effect in vivo.

**Results:**

Compared with cisplatin or oleandrin alone, the combined treatment significantly inhibited cell proliferation and induced cell apoptosis. The median-effect analysis indicated a synergistic cytotoxic effect. The combined treatment downregulated Bcl-2 and upregulated Bax and cleaved caspase-3, -8 and -9. And the suppression of caspases reduced cell death. Furthermore, oleandrin alone or with cisplatin, activated the p38 MAPK/Elk-1 pathway. The inhibition of the p38 MAPK pathway increased cell viability and reduced apoptosis. In vivo, the combined treatment was also verified to significantly inhibit tumor growth, induce apoptosis and activate the p38 MAPK pathway.

**Conclusions:**

The combination of oleandrin with cisplatin exerts a synergistic antitumor effect in osteosarcoma, which relates to the activation of the p38 MAPK pathway.

## Introduction

Osteosarcoma is the most frequent primary malignant bone tumor and predominantly affects young adults and adolescents [1]. The 5-year overall survival rate has increased from < 20% with surgery alone to 60–65% with the addition of chemotherapy [2]. However, recent decades have witnessed no further improvements in survival. In addition, drug-induced side effects and resistance limit the application of conventional chemotherapy [3]. Therefore, novel therapeutic agents and strategies with high potency are urgently needed.

Oleandrin (OLE), one type of polyphenolic cardiac glycoside extracted from the leaves of *Nerium oleander* L., has long been used to treat congestive heart failure by inhibiting Na^+^/K^+^-ATPase [4]. OLE exerts antitumor effects on several types of human tumor cells, including prostate adenocarcinoma, pancreatic cancer and glioma [5–7], but has no significant cytotoxicity against non-malignant cells [8]. Our previous studies reported that OLE has antitumor activity against osteosarcoma in vitro and does not induce the death of normal hFOB1.19 human osteoblast cells [9, 10]. These findings suggest that OLE may be an effective drug therapy for osteosarcoma.

Cisplatin (DDP), a first-line chemotherapeutic drug, is widely used to treat various tumors, including osteosarcoma. However, drug resistance and its main side effect of nephrotoxicity severely limit its therapeutic effect [3]. Recently, accumulating studies have demonstrated that OLE can potentially sensitize tumor cells to DDP. In two colon cancer cell lines HT29 and HCT116, the combination of OLE with DDP or oxaliplatin had additive or synergistic inhibitory effects [11]. In A549 human lung cancer cells, an extract from the leaves of *N. oleander* showed moderate synergism when administered after DDP [12]. The combination of Anvirzel, a *N. oleander* extract that is mainly composed of OLE and its deglycosylated metabolite oleandrigenin, with DDP exerted a synergistic effect in various cancer cells [13]. However, no report has revealed whether OLE increases the sensitivity of osteosarcoma cells to DDP.

p38 mitogen-activated protein kinase (MAPK) pathway, a key member in the MAPK superfamily, regulates a variety of cellular responses to stress and inflammation. Two major groups of substrate regulated by p38 MAPK phosphorylation are transcription factors, such as p53, activating transcription factor 2 (ATF2), myocyte-specific enhancer factor 2 (MEF2) and ETS transcription factor (Elk-1); and protein kinases like MAPK-activated kinase 2 (MK2) [14]. Evidence suggests that the suppression of the p38 MAPK pathway relates to the development of DDP resistance [15, 16]. The cardiac glycosides bufalin and ouabain have been shown to activate p38 MAPK in human umbilical vein endothelial cells (HUVECs) and breast cancer cells, respectively [17, 18]. Whether OLE activates p38 MAPK is unknown, and whether the potential activation of p38 MAPK is involved in the combined effect of DDP and OLE in osteosarcoma needs to be clarified.

The present study had the following aims: (1) to determine the potential synergistic antitumor effect of DDP and OLE on osteosarcoma cells in vitro; (2) to explore the underlying mechanisms involved in the combined effect; and (3) to further evaluate the combined effect on osteosarcoma growth in vivo.

## Materials and methods

### Reagents and antibodies

The following reagents and antibodies were used in this study: OLE, SB203580 (Sigma-Aldrich Chemical Co., St. Louis, MO, USA); DDP (Jiangsu Hanson Pharmaceutical Ltd., Jiangsu, China); Z-VAD-FMK (Beyotime Biotech Ltd., Nanjing, China); antibodies against B-cell lymphoma protein 2 (Bcl-2), Bcl-2-associated X protein (Bax), cleaved caspase-3, cleaved caspase-8, cleaved caspase-9, phospho-p38 MAPK (P-p38), p38 MAPK, phospho-Elk-1 (P-Elk-1) and Elk-1 (Cell Signaling Technology, Beverly, MA, USA); and antibody against β-actin (CWBIO Biotech Ltd., Beijing, China).

### Cell culture

The MG-63 and 143B cells were obtained from the American Type Culture Collection (ATCC, Manassas, VA, USA). The source and culture method of U-2OS cells were described previously [9]. The human kidney-2 (HK-2) immortalized human proximal tubular cells were kindly provided by Dr. Yue Wang from Peking University Third Hospital. MG-63 and 143B cells were cultured in Dulbecco’s modified Eagle’s medium (DMEM)/high glucose, and HK-2 cells were cultured in DMEM/F-12 medium (HyClone, Logan, UT, USA). All media contained 10% fetal bovine serum (FBS) (Gibco, Grand Island, NY, USA) and 1% penicillin–streptomycin (10,000 U/mL) (Gibco). The cells were incubated at 37 °C in a humidified 5% CO_2_ incubator.

### Cell viability assay

Five thousand cells per well were seeded into 96-well plates, incubated overnight and then treated with DDP and/or OLE for 24 h. Then, the cells were incubated with 10 µL of cell counting kit-8 (CCK-8) reagent (Dojindo Laboratories, Kumamoto, Japan) for 2 h. An automatic ELISA plate reader was used to measure the absorbance at 450 nm. The surviving fraction was calculated as follows: (%) = [OD (treatment)-OD (blank)]/[OD (control)-OD (blank)] × 100%. The experiments were repeated three times with at least triplicate wells for each concentration.

### Combination analysis

The combined effect of DDP and OLE was evaluated based on the median-effect equation proposed by Chou and Talalay [19] and quantified using the combination index (CI), where CI < 1, CI = 1 and CI > 1 indicate synergy, an additive effect and antagonism, respectively. The median-effect equation: fa/fu = [*D*/Dm]^*m*^, where *D* is the concentration of a drug, fa is the cell fraction affected by *D* (i.e., percentage inhibition/100), and fu is the fraction unaffected (i.e., 1 − fa), Dm is the median-effect dose that inhibits cells under study by 50%, and *m* is the coefficient signifying the shape of the dose–effect relationship. The values of fa were obtained from CCK-8 assays, where cells were treated with OLE, DDP and OLE + DDP at fixed ratios according to the equation. Then, putting fa values into the median-effect equation can generate the corresponding doses of cisplatin/oleandrin alone or in combination at any fa. Finally, for each fa, a corresponding CI was produced as follows: CI = (*D*)_1_/(*D*_f_)_1_ + (*D*)_2_/(*D*_f_)_2_ + (*D*)_1_(*D*)_2_/(*D*_f_)_1_(*D*_f_)_2_, where (*D*)_1_ and (*D*)_2_ are the doses of drug 1 combined with drug 2 that are needed to produce fa, and (*D*_f_)_1_ and (*D*_f_)_2_ are the doses of the drugs alone to produce the same fa. In cancer treatment, a combination is explored to achieve maximal effects; therefore, fa < 0.5 was deemed irrelevant. All these calculating operations were performed with the CalcuSyn software version 2.1 (BIOSOFT, Great Shelford, Cambridge, UK).

### Colony formation assay

Two thousand cells per well were plated into 6-well plates and allowed to adhere for 24 h. Then, the cells were treated with control medium, DDP, OLE or DDP + OLE for 10 days. After incubation, the colonies were washed with 1 × phosphate-buffered saline (PBS), fixed with 4% ice-cold paraformaldehyde for 15 min and stained with 0.1% crystal violet, and the colony numbers were counted manually. Then, the dye was washed away, and the number of colonies that contained more than 50 cells was determined.

### 4′,6-Diamidino-2-phenylindole (DAPI) staining

The cells were seeded in 24-well plates and treated as indicated above for 24 h. After treatment, the cells were washed with 1 × PBS and fixed with 4% ice-cold paraformaldehyde for 15 min. Then, the cells were incubated with a DAPI staining solution (Beyotime) for 5 min at room temperature in the dark. A fluorescence microscope (Leica DM3000, Frankfurt, Germany) was used to observe and image cell nuclei at a 100 × magnification.

### Annexin V-FITC/propidium iodide (PI) apoptosis assay

The cells were seeded into six-well plates and adhered overnight. Then, the cells were treated as described above. Subsequently, the cells were trypsinized and harvested in 1 × PBS. According to the protocol in the Annexin V-FITC Apoptosis Detection Kit (BD Biosciences, Franklin Lakes, NJ, USA), the cells were suspended in 1 × Annexin V binding buffer and stained with Annexin V-FITC and PI at room temperature for 5 min in the dark. Finally, the cells were analyzed by a CytoFLEX flow cytometer (Beckman Coulter, Brea, CA, USA).

### Western blotting assay

The cells were lysed using the Total Protein Extraction Kit (Applygen Technologies Inc., Beijing, China), and the BCA Protein Assay Kit (Applygen Technologies Inc.) was applied to measure protein concentrations. Equal amounts of protein samples were separated by sodium dodecyl sulfate polyacrylamide gel electrophoresis (SDS–PAGE) and transferred to polyvinylidene fluoride (PVDF) membranes. Then, the membranes were blocked with 5% bovine serum albumin at room temperature for 1 h and incubated with the primary antibodies overnight at 4 °C. The next day, after washing with Tris-buffered saline with Tween 20 (TBST), the membranes were incubated with horseradish peroxidase (HRP)-conjugated secondary antibodies at room temperature for 1 h. The proteins were visualized by enhanced chemiluminescence (ECL) (Millipore Corp., Billerica, MA, USA) according to the manufacturer’s protocol.

### Nude mouse xenograft studies

BALB/c nu/nu mice (Beijing HFK Bio-Technology Co., Ltd, Beijing, China) were maintained under specific pathogen-free conditions in the Department of Laboratory Animal Science of Peking University Health Science Center. Experimental procedures involving the animals were approved by the Peking University Institutional Review Board and performed in accordance with the approved guidelines. A total of 1 × 10^6^ 143B cells were suspended in 100 µL of PBS and injected subcutaneously into the dorsal right flank of BALB/c nu/nu mice (aged 4 weeks and weighing approximately 20 g). When the tumor volume reached approximately 150 mm^3^, the mice were randomly divided into four groups (six mice/group) and treated with drugs by intraperitoneal injection every two days as follows: (1) control group (physiological saline, 100 µL); (2) DDP group (1.5 mg/kg body weight); (3) OLE group (0.3 mg/kg body weight); and (4) DDP + OLE group (DDP, 1.5 mg/kg body weight and OLE, 0.3 mg/kg body weight). The animals were treated for 16 days. The body weight and tumor size were measured every 3 days, and the tumor volumes were calculated by the following formula: *L* × *W*^2^/2 (*L*, long diameter of the tumor; *W*, short diameter of the tumor).

### Terminal deoxynucleotidyl transferase (TdT)-mediated dUTP nick end labeling (TUNEL) staining

A TUNEL assay kit (Roche Diagnostics, Mannheim, Germany) was used to detect DNA fragmentation according to the manufacturer’s instructions. Briefly, the sections were deparaffinized, hydrated and incubated with proteinase K for 15 min at room temperature, followed by incubation with the TUNEL reaction mixture. TdT labeling was visualized by an HRP-conjugated Fab fragment.

### Immunohistochemical staining

The xenografts were excised after the mice were killed and used to prepare formalin-fixed paraffin-embedded tissue sections. An EnVision two-step staining method was performed. Briefly, after dewaxing in xylene and rehydration in a gradient of ethanol concentrations before antigen retrieval, the sections were incubated with a caspase-3 (1:1000) antibody overnight at 4 °C. Then, the sections were washed with PBS and incubated with an IgG-HRP polymer (CWBIO) and the diaminobenzidine substrate. Staining was scored according to the product of the intensity (0—no staining; 1—weak; 2—moderate; and 3—strong) and the percentage of positive staining (0, 0%; 1, 25%; 2, 26–50%; 3, 51–75%; and 4, 76–100%). The expression level of caspase-3 was defined as −, +, ++ and +++ based on the median staining score.

### Statistical analysis

The data were analyzed by IBM SPSS Statistics 20.0 software (IBM, Armonk, NY, USA), and the results are expressed as the mean ± standard deviation (SD). The statistical analyses of the cell surviving fraction, half maximal inhibitory concentration (IC_50_), CI and DRI were performed using Student’s *t* test. The statistical analyses for colony formation and apoptosis rates were conducted using one-way analysis of variance (ANOVA), followed by Dunnett’s *t* test to compare multiple treatment groups with the control group or the combined group. Differences were considered significant at *p* < 0.05.

## Results

### The combination of DDP with OLE promotes cytotoxicity in osteosarcoma cells but not kidney cells

**Fig. 1:**
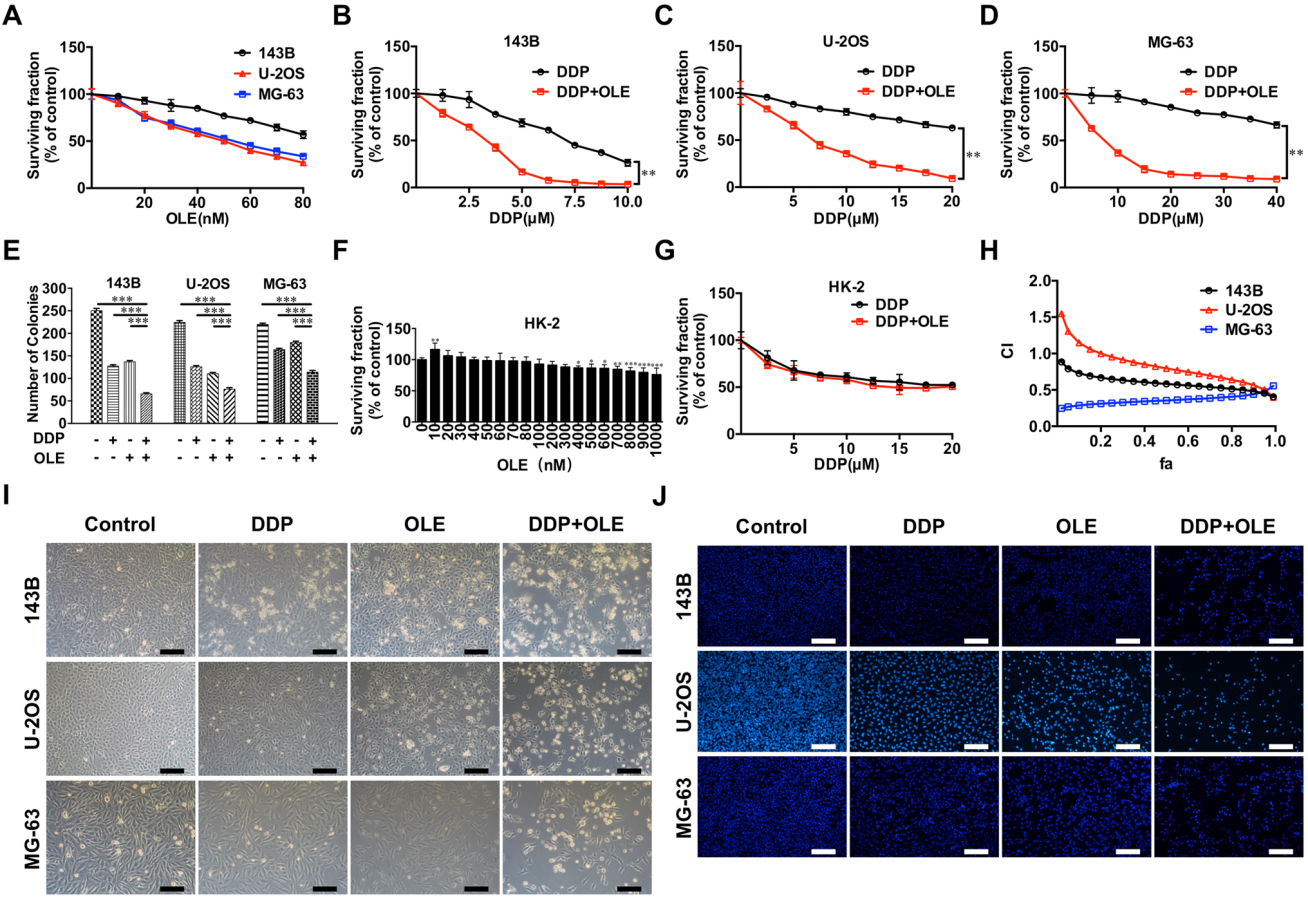
The cytotoxic effects of oleandrin (OLE) and/or cisplatin (DDP). **a** The surviving fractions of 143B, U-2OS and MG-63 osteosarcoma cells after 24 h of OLE treatment at continuous concentrations (0–80 nM). **b**–**d** The surviving fractions of 143B, U-2OS and MG-63 cells after treatment with continuous concentrations of DDP (143B, 0–10 µM; U-2OS, 0–20 µM; MG-63, 0–40 µM) and DDP + OLE for 24 h at fixed ratios (143B, 1:125; U-2OS, 1:250; and MG-63, 1:500). **e** The number of colonies in 143B, U-2OS and MG-63 cells after 24 h of exposure to DDP and/or OLE (143B, DDP 5 µM; U-2OS, DDP 10 µM; MG-63, DDP 20 µM; OLE 40 nM). **f** The surviving fractions of HK-2 normal human kidney cells after exposure to OLE (0–1000 nM) for 24 h. **g** The surviving fraction of HK-2 cells after treatment with DDP and DDP + OLE for 24 h (DDP: 0–20 µM, OLE: 0–80 nM). **h** The combination index (CI) values calculated from each fraction affected (fa) post-treatment. **i** Representative phase contrast images showing changes in cell morphologies in the three osteosarcoma cell lines after treatment as above. **j** Representative fluorescence microscopy images showing changes in cell nuclei in the three osteosarcoma cell lines after treatment as above. Data are presented as the mean ± SD. Scale bar 100 µm. *p < 0.01, **p < 0.01 and ***p < 0.001

To determine the cytotoxic effects of the drugs on cell viability, CCK-8 assays were conducted. As shown in Fig. 1a and Table 1, U-2OS and MG-63 cells were more sensitive to OLE than 143B cells (the IC_50_ values of OLE for U-2OS, MG-63 and 143B cells at 24 h were 45.84 ± 1.02, 51.55 ± 1.73 and 103.57 ± 4.48 nM, respectively). Conversely, U-2OS and MG-63 cells were more resistant to DDP than 143B cells (the IC_50_ values of DDP for 143B, U-2OS and MG-63 cells at 24 h were 6.98 ± 0.33, 30.47 ± 1.47 and 62.81 ± 2.63 µM, respectively) (Fig. 1b–d; Table 1). To explore whether OLE enhances the cytotoxicity of DDP, the cells were simultaneously and continuously exposed to increasing concentrations of OLE and DDP at fixed ratios (143B, 1:125; U-2OS, 1:250; and MG-63, 1:500). Take the ratio of 1:125 in 143B cells as an example, it means OLE at a concentration of 10 nM must combine with DDP at a corresponding concentration of 1.25 µM, and OLE at a concentration of 20 nM must combine with DDP at a corresponding concentration of 2.5 µM, and so on. The results revealed that the combined treatment significantly inhibited cell viability in the three cell lines (all *p* < 0.01; Fig. 1b–d). Quantitatively, the combined treatment reduced the IC_50_ values of DDP to 2.61 ± 0.05, 6.61 ± 0.40 and 6.40 ± 0.80 µM in 143B, U-2OS and MG-63 cells, respectively (DDP group vs. DDP + OLE group, all *p* < 0.01; Table 1). The IC_50_ values of OLE also exhibited significant reductions to 20.88 ± 0.41, 26.46 ± 1.61 and 12.80 ± 1.59 nM, respectively (OLE group vs. DDP + OLE group, all *p* < 0.001; Table 1). In addition, we performed colony formation assays to assess the effects of DDP and/or OLE on cell proliferation. The results showed that the combined treatment caused significant reductions in the number of colonies compared with the results obtained for DDP or OLE alone (DDP group or OLE group vs. DDP + OLE group, all *p* < 0.001; Fig. 1e).

**Table 1:** Growth inhibition by DDP and/or OLE in osteosarcoma cells

Cell line	IC_50_ of DDP (µM)		*p* value	IC_50_ of OLE (nM)		*p* value
	Alone	Combination		Alone	Combination	
143B	6.98 ± 0.33	2.61 ± 0.05	0.002	103.57 ± 4.48	20.88 ± 0.41	< 0.001
U-2OS	30.47 ± 1.47	6.61 ± 0.40	< 0.001	45.84 ± 1.02	26.46 ± 1.61	< 0.001
MG-63	62.81 ± 2.63	6.40 ± 0.80	< 0.001	51.55 ± 1.73	12.80 ± 1.59	< 0.001

Severe nephrotoxicity is a major obstacle that limits the therapeutic effect of DDP. Therefore, we evaluated the cytotoxicity of OLE in HK-2 normal human kidney cells. OLE did not induce a significant loss of HK-2 cell viability at concentrations up to 400 nM, which is fourfold higher than the highest dose we used in osteosarcoma cells (Fig. 1f). Furthermore, the addition of OLE to DDP did not cause a significant difference in cell viability compared with the value obtained for DDP alone (Fig. 1g).

### The combination of DDP with OLE exerts a synergistic antitumor effect in osteosarcoma cells

To assess whether the combined effect of DDP and OLE represented synergism, we calculated the CI values based on the median-effect equation. The fa-CI plot demonstrated that the CI values were all lower than 1 (0.4–0.8) in the three osteosarcoma cell lines when fa ≥ 0.50, suggesting a synergistic antitumor effect of DDP and OLE (CI values at fa_0.50_ vs. 1.00, all *p* < 0.05; CI values at fa_0.75_ vs. 1.00, all *p* < 0.01; and CI values at fa_0.90_ vs. 1.00, all *p* < 0.01; Fig. 1h; Table 2). CI values of 0.51, 0.73 and 0.43 were obtained from the combination of 40 nM OLE with 5 µM DDP in 143B cells, 10 µM DDP in U-2OS cells and 20 µM DDP in MG-63 cells, respectively. We selected these combination doses for further studies.

**Table 2:** The combination index (CI) values for the combination of DDP with OLE in osteosarcoma cells

Cell line	CI at fa_0.50_	CI at fa_0.75_	CI at fa_0.90_	Interpretation
143B	0.58 ± 0.01***	0.50 ± 0.01***	0.44 ± 0.01***	Synergism
U-2OS	0.80 ± 0.07*	0.67 ± 0.03**	0.58 ± 0.02**	Synergism
MG-63	0.35 ± 0.05**	0.38 ± 0.04**	0.42 ± 0.04**	Synergism

^†^*p < 0.05, **p < 0.01, and ***p < 0.001 (one-sample, two-tailed *t* test compared with 1.00)

### The combination of DDP with OLE enhances apoptosis in osteosarcoma cells

**Fig. 2:**
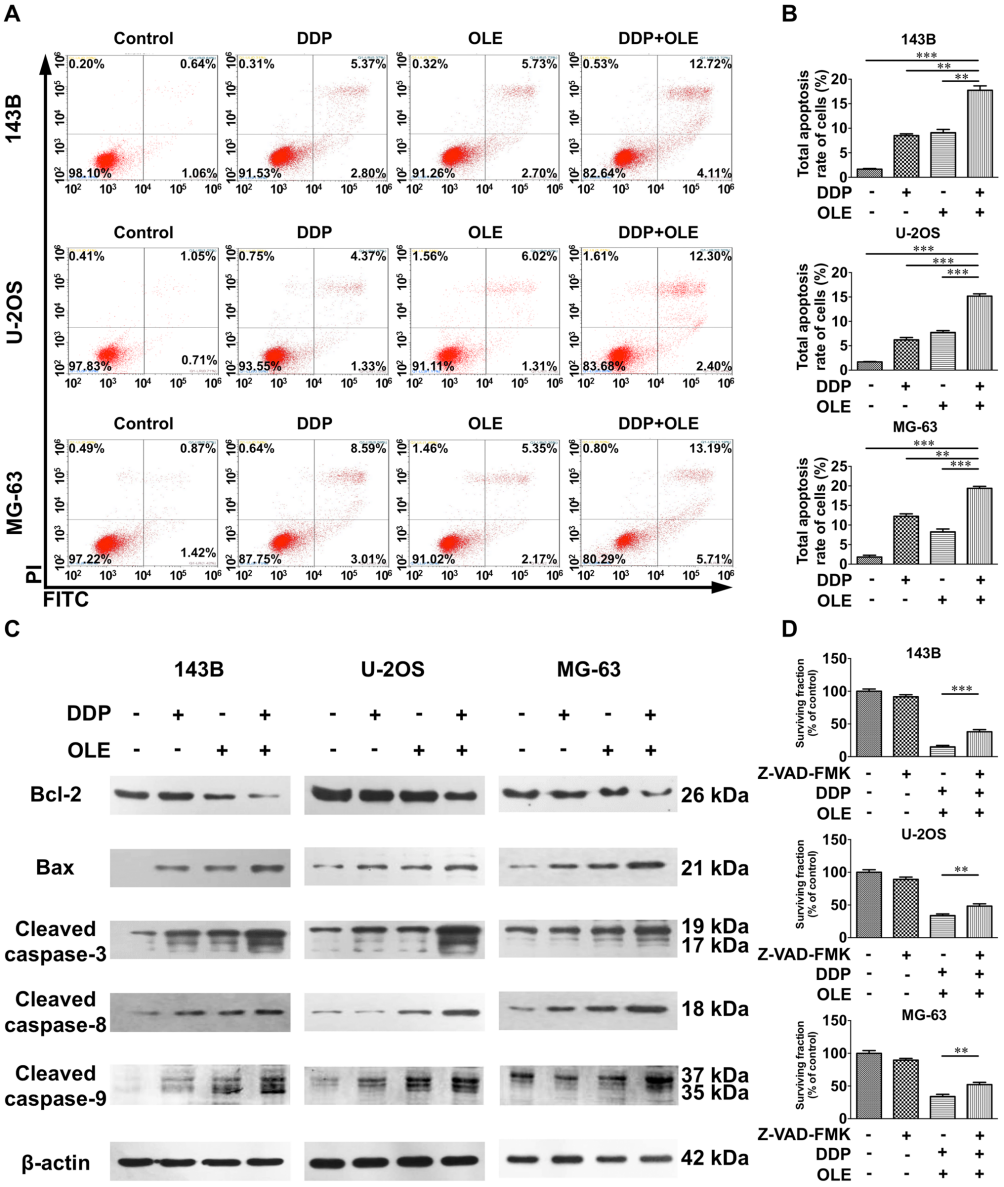
Cell apoptosis induced by cisplatin (DDP) and/or oleandrin (OLE). **a** The apoptosis rates of 143B, U-2OS and MG-63 cells after treatment with DDP and/or OLE for 24 h (143B, DDP 5 µM; U-2OS, DDP 10 µM; MG-63, DDP 20 µM; OLE 40 nM). **b** The quantitative results of the apoptosis analysis of 143B, U-2OS and MG-63 cells after treatment as above. **c** The expression of Bcl-2, Bax and cleaved caspase-3, 8 and 9, as detected by western blots after treatment as above. **d** The effects of pretreatment (for 1 h) with a pan-caspase inhibitor Z-VAD-FMK (20 µM) on the cell viability of the DDP + OLE-treated (for 24 h) osteosarcoma cells. Data are presented as the mean ± SD. **p < 0.01 and ***p < 0.001

DDP exerts cytotoxicity mainly through inducing apoptosis. Next, we tested whether the enhanced cytotoxicity of the combined therapy could be attributed to cell apoptosis. Figure 1i shows the cell shrinkage after exposure to the monotherapies and the combined therapy; the combined group exhibited the most significant morphological changes. DAPI staining showed the appearance of chromatin condensation and DNA fragmentation in all three treatment groups. Similar to the morphological changes, cells exposed to the combined treatment appeared to exhibit more severe DNA damage (Fig. 1j). In addition, flow cytometry analysis showed that the apoptosis rate of 143B cells in the combined group was 17.74%, which was significantly higher than the 1.67% observed in the control group, 8.51% in the DDP group and 9.10% in the OLE group (control group, DDP group and OLE group vs. combined group, all *p* < 0.01; Fig. 2a, b). The apoptosis rates of U-2OS cells in the control group, DDP group, OLE group and combined group were 1.69%, 6.19%, 7.72% and 15.18%, respectively (control group, DDP group and OLE group vs. combined group, all *p* < 0.001; Fig. 2a, b). Additionally, the apoptosis rates of MG-63 cells in the above four groups were 1.83%, 12.23%, 8.26% and 19.40%, respectively (control group, DDP group and OLE group vs. combined group, all *p* < 0.01; Fig. 2a, b).

### The combination of DDP with OLE increases the activation of both intrinsic and extrinsic apoptotic pathways in osteosarcoma cells

There are two main apoptotic pathways induced by cytotoxic agents: the intrinsic and extrinsic pathways. We detected apoptosis-related proteins, including Bcl-2, Bax, and caspase-3, 8 and 9 to determine which pathway was induced by the combined therapy. Caspase-3 is the common executive caspase in both apoptotic pathways, while Bcl-2, Bax and caspase-9 are involved in the intrinsic apoptotic pathway, and caspase-8 plays a role in the extrinsic pathway. The results clearly indicated that the monotherapies and the combined therapy all activated caspase-3 and -8 and triggered the expression of Bax (Fig. 2c). Moreover, OLE and the combined treatment also obviously caused the upregulation of cleaved caspase-9 and the downregulation of Bcl-2, whereas DDP alone only slightly increased the activation of caspase-9 in 143B and U-2OS cells and did not alter the expression of Bcl-2 in any osteosarcoma cells (Fig. 2c). In terms of the expression levels of these proteins, the combined treatment significantly increased the levels of Bax and cleaved caspase-3, -8 and -9 and obviously decreased the level of Bcl-2 compared with the values observed for DDP or OLE alone (Fig. 2c). To confirm the role of cell apoptosis in the combined therapy, we added Z-VAD-FMK (a pan-caspase inhibitor) at a dose of 20 µM to pretreat combined group and found that cell viability was significantly enhanced (Fig. 2d).

### OLE alone or in combination with DDP activates p38 MAPK and Elk-1

**Fig. 3:**
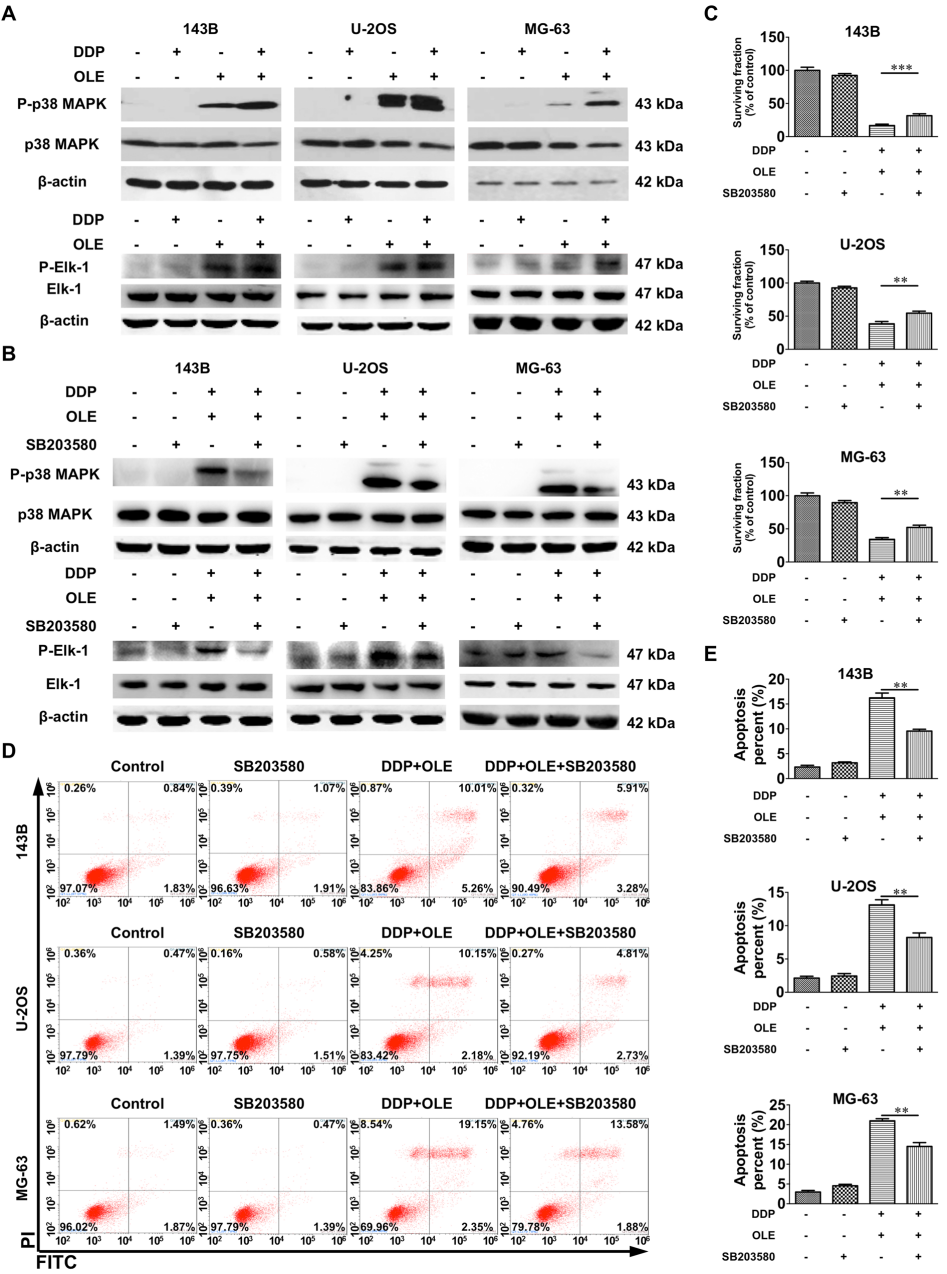
The influence of cisplatin (DDP) and/or oleandrin (OLE) on the activation of the p38 MAPK pathway. **a** The expression of phospho-p38 MAPK (P-p38 MAPK) and phospho-Elk-1 (P-Elk-1) detected by western blot after treatment with DDP and/or OLE for 24 h (143B, DDP 5 µM; U-2OS, DDP 10 µM; MG-63, DDP 20 µM; OLE 40 nM). **b** The expression of P-p38 MAPK and P-Elk-1 after 1 h of a p38 MAPK inhibitor SB203580 (143B, 2 µM; U-2OS and MG-63, 10 µM) pretreatment and subsequent 24 h of DDP + OLE treatment at the above concentrations. **c** Effects of SB203580 on the cell viability of DDP + OLE-treated osteosarcoma cells. **d, e** The apoptosis rates and quantitative results of the apoptosis analysis after 1 h of SB203580 pretreatment, followed by 24 h of DDP + OLE treatment as above. *n* = 3. Data are presented as the mean ± SD. **p < 0.01 and ***p < 0.001

The p38 MAPK pathway plays a key role in the process of DDP-induced apoptosis. Western blot assays showed that OLE and the combined treatment activated p38 MAPK, while no activation of p38 MAPK was found after DDP treatment alone (Fig. 3a). Elk-1 is a transcription factor that can be activated by P-p38 MAPK. As shown in Fig. 3a, OLE alone or combined with DDP also upregulated the expression of P-Elk-1. Further, to verify the role of the p38 MAPK pathway in the enhanced apoptosis, cells were pretreated with the p38 MAPK inhibitor SB203580 (143B, 2 µM; U-2OS and MG-63, 10 µM) and then exposed to DDP + OLE treatment. The P-p38 MAPK expression was obviously suppressed (Fig. 3b) and the enhanced expression of P-Elk-1 in combined groups was also reduced by SB203580 (Fig. 3b). Meanwhile, CCK-8 assays showed that SB203580 significantly attenuated the cell cytotoxicity of the combined treatment in osteosarcoma cells (Fig. 3c). In addition, SB203580 notably reversed the apoptosis caused by the combined treatment (DDP + OLE group vs. DDP + OLE + SB203580 group in 143B, U-2OS and MG-63 cells, all *p* < 0.01; Fig. 3d, e).

### OLE potentiates the antitumor activity of DDP in the osteosarcoma xenograft model

**Fig. 4:**
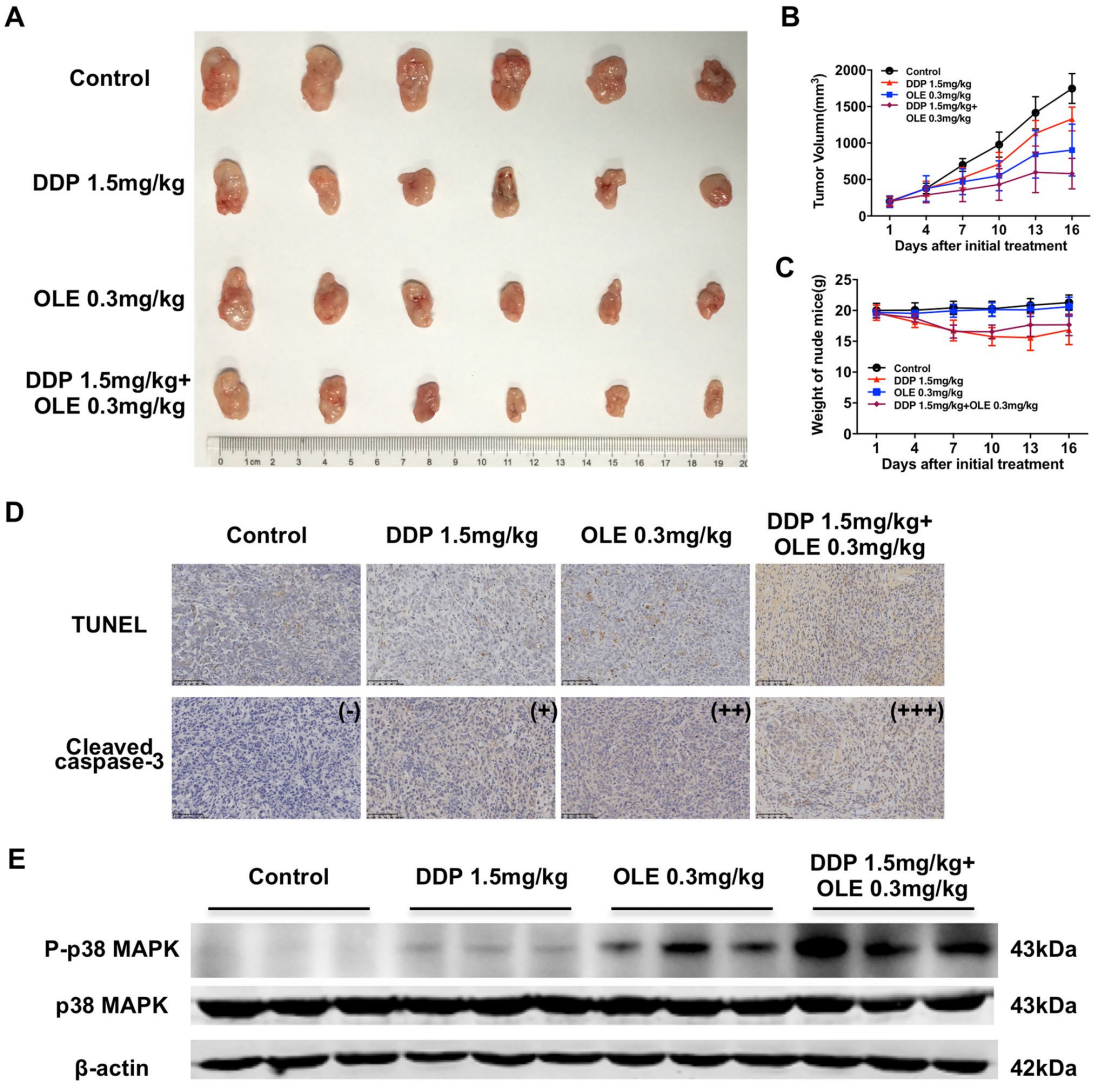
The inhibition effects of cisplatin (DDP) and/or oleandrin (OLE) on 143B osteosarcoma xenografts. **a** The photograph of excised osteosarcoma xenografts after intraperitoneal injections of physiological saline, DDP (1.5 mg/kg), OLE(0.3 mg/kg) and DDP (1.5 mg/kg) + OLE (0.3 mg/kg) every two days for 16 days. **b** Tumor volumes within 16 days after the initial treatment. **c** The weight of the nude mouse within 16 days after the initial treatment. **d** Representative images of the tumor xenografts stained for DNA fragmentation by a TUNEL assay and immunohistochemistry staining for cleaved caspase-3. **e** The expression level of phospho-p38 MAPK (P-p38 MAPK) of tumor xenografts. Scale bar 100 µm

To confirm the in vitro findings, we further examined the in vivo effect of DDP and/or OLE. As shown in Fig. 4a, b, compared with the results obtained for the physiological saline treatment, the DDP treatment and the OLE treatment significantly suppressed the growth of the xenograft tumors (DDP group or OLE group vs. physiological saline group, all *p* < 0.01). The combination of DDP with OLE showed more efficient tumor suppression than DDP or OLE alone (physiological saline group, DDP group or OLE group vs. DDP + OLE group, *p* < 0.001, *p* < 0.001 and *p* < 0.05, respectively; Fig. 4a, b). In addition, DDP obviously reduced the weight of the mice, while OLE did not cause significant weight loss and DDP + OLE did not increase weight loss than DDP alone (Fig. 4c). TUNEL assays of xenograft tumors revealed more cell death in the DDP + OLE group than in the DDP or OLE group (Fig. 4d). Immunohistochemical quantitative analysis showed that the expression level of cleaved caspase-3 was higher in the DDP + OLE group than in the DDP or OLE group (Fig. 4d). And the western blot assay verified the activation of P-p38 MAPK *in vivo* after OLE or DDP + OLE treatment (Fig. 4e).

## Discussion

The survival rate of osteosarcoma has reached a plateau in recent decades. Our previous studies found that OLE exerted antitumor effects on osteosarcoma cells by inhibiting the Wnt/β-catenin signaling pathway [9, 10]. In the present study, we demonstrated that OLE synergistically enhanced the inhibitory effect of DDP against osteosarcoma cells in vitro and in vivo by promoting cell apoptosis. In addition, OLE showed no obvious toxicity in normal human kidney cells or nude mice. These findings suggest that the combination of DDP with OLE could be a novel, reliable and safe strategy for osteosarcoma treatment.

Drug combinations are commonly used in treating cancer to achieve better therapeutic effects and less side effects. Our study offers the evidence that DDP combined OLE achieves a synergistic effect in osteosarcoma. Similarly, Felth et al. reported that OLE combined with DDP or oxaliplatin had additive or synergistic antitumor effects in colon cancer cells [11]. Our finding is also consistent with previous studies showing that the extract from *N. oleander*, a mixture of OLE and other ingredients, sensitizes multiple kinds of cancer cells, including breast, lung, colon, prostate, melanoma and pancreatic cancer, to DDP in a synergistic manner [12, 13].

DDP exerts cytotoxicity mainly by triggering cell apoptosis [20]. In embryonal carcinoma and lung cancer, drugs sensitize tumor cells to DDP by enhancing DDP-induced apoptosis [21, 22]. Two main apoptotic pathways are involved in the cytotoxic agents-mediated apoptosis: the intrinsic and extrinsic pathways. In the intrinsic pathway, the proteins of the Bcl-2 family, including the anti-apoptotic proteins Bcl-2, Bcl-x and Bcl-XL and the pro-apoptotic proteins Bax, Bak and Bim, control mitochondrial membrane permeability and the subsequent release of cytochrome *c* from mitochondria. Then, cytochrome *c* binds Apaf-1 and procaspase-9 to form the apoptosome, which activates caspase-9 and caspase-3 to induce apoptosis [23]. In the extrinsic pathway, extracellular apoptotic signals are delivered to caspase-8 in the form of ligands binding to their death receptors (DRs), such as FasL/Fas, TNF/TNFR and Apo3L/DR3. Then, the activation of caspase-8 initiates apoptosis by directly cleaving caspase-3 or continuing to activate the intrinsic apoptotic pathway [24]. Our observations showed that OLE alone and the combined treatment activated both the intrinsic and extrinsic apoptotic pathways, whereas DDP chiefly triggered the extrinsic apoptotic pathway. Similarly, DDP is widely known to mainly activate caspase-8 and -3 to induce the extrinsic apoptotic pathway in tumor cells [21, 25] and OLE was demonstrated to induce both apoptotic pathways [10]. Our study demonstrated that the addition of OLE to DDP remarkably decreased the Bcl-2/Bax ratio and involved various caspases functioning together, thus enhancing cell apoptosis. And the distinct cell rescue effect by pan-caspase inhibitor Z-VAD-FMK in the combined group further verified the role of cell apoptosis in the combined effect of DDP and OLE.

Many factors act together to influence the sensitivity of cancer cells to DDP, including DDP uptake, DDP detoxication, repair of DNA damage, tolerance to unrepaired DNA and activity in apoptotic signal pathways [26]. Evidence has shown that the activation of the p38 MAPK pathway plays a role in the DDP-induced apoptosis [20]. Here we first reported that OLE activated the phosphorylation of p38 MAPK and found the phospho-p38 MAPK was involved in the enhanced apoptosis caused by combined treatment. As a type of stress-activated protein kinase pathway, the activation of the p38 MAPK pathway induces apoptosis in osteosarcoma, hepatocellular carcinoma and breast cancer [27–30]. Brozovic et al. found that human cervical carcinoma cells with DDP resistance displayed an apparently reduced activation of p38 MAPK [15]. Zhang et al. reported that the suppression of p38 MAPK activation by heat shock protein 27 (Hsp27) endowed mouse fibroblast cells with DDP resistance [16]. These studies suggested that p38 MAPK pathway might contribute to DDP resistance. We observed that DDP alone did not cause activation of p38 MAPK in osteosarcoma cells, indicating that the osteosarcoma cells were resistant to DDP at this dose. We observed that DDP + OLE induced the activation of p38 MAPK and the P-p38 MAPK inhibition by SB203580 reduced the cytotoxicity of the two drugs, suggesting that the p38 MAPK pathway played a partial role in the combined effects. In line with our findings, Weir et al. reported that curcumin induced apoptosis in cisplatin-resistant ovarian cancer cells through the activation of p38 MAPK and p53 [31]. Tripathi et al. found that the addition of fisetin, a flavonoid, to cisplatin increased the phosphorylation of p38 MAPK in human embryonal carcinoma cells and that the inhibition of p38 MAPK phosphorylation resulted in significant protection for the cells [21].

The phosphorylation of p38 MAPK further activates the downstream proteins to transmit signals. As a downstream substrate of phospho-p38 MAPK, Elk-1 is a transcription factor belongs to the ETS-domain family and involved in cell apoptosis. In squamous cell carcinoma, the inhibitor of DNA binding 3 (Id3) induced apoptosis through an Elk-1-caspase-8-dependent pathway [32]. In HaCaT Keratinocytes, the activation of Elk-1 mediated sodium arsenite-induced apoptosis by upregulating Bax expression [33]. In addition, Elk-1 has been reported to upregulate the expression of death receptor 5 (DR5), a transmembrane receptor that triggers the extrinsic apoptotic pathway, in breast cancer and lung cancer [34, 35]. In our study, we found that the combined treatment enhanced the phosphorylation of Elk-1 and the induction of SB203580 into combined treatment attenuated the activation of Elk-1, suggesting Elk-1 was a key transcription factor influenced by P-p38 MAPK in the process of DDP + OLE-induced cell apoptosis.

Our in vivo data showed that the combined therapy exhibited more efficient tumor suppression than did the monotherapies. Similarly, OLE combined with temozolomide significantly improved survival time in glioma-bearing mice compared with temozolomide alone [7]. However, we found the combined effect was weaker in vivo than in vitro. The drug ratio plays a critical role in getting ideal therapeutic efficacy in combination studies. However, not like in the in vitro study, it is hard to find an appropriate ratio in the in vivo study, which is time-consuming and costly [36]. Meanwhile, variable drug pharmacokinetic profiles further increase the difficulty to keep a satisfying drug ratio [37]. In our study, the modest combined effect in vivo may result from the less-than-ideal ratio of DDP and OLE. Our further in vivo studies need to set a better drug ratio according to the in vitro data and the pharmacokinetics of DDP and OLE.

Our previous studies reported that OLE did not induce the death of hFOB1.19 normal human osteoblast cells [10]. In this study, we found that OLE alone only had a minor inhibiting effect on HK-2 normal human kidney cells at high doses and OLE combined DDP did not increase cytotoxicity in HK-2 cells compared to DDP alone. This finding is consistent with a previous study of the pharmacokinetics and metabolism of OLE in CD-1 mice, which demonstrated that the kidney retained considerably little of OLE after intravenous injection [38]. Moreover, our in vivo study showed that OLE did not reduce the weight of nude mice, while not increased the toxicity when combined with DDP. Similar to our findings, Sreenivasan et al. reported that OLE induced apoptosis in human tumor cells but not in normal cells, such as neutrophils [39]. Calderón-Montaño et al. observed that MRC5 human lung nonmalignant cells were 10-fold resistant to OLE than A549 lung cancer cells [12]. The selectivity of OLE may arise from the relative expression of the Na^+^/K^+^-ATPase α1 and α3 subunits [8], which should be further explored in osteosarcoma.

In summary, we conclude that the combination of DDP with OLE exerts a synergistic antitumor effect in osteosarcoma in vitro and in vivo. This synergistic effect mainly depends on enhanced cell apoptosis, which partly results from the activation of the p38 MAPK/Elk-1 pathway. Further studies are needed to achieve better therapeutic effects in vivo and to investigate the potential selective mechanisms of OLE between osteosarcoma cells and normal cells.
